# Ideal Adsorbed Solution Theory (IAST) of Carbon Dioxide and Methane Adsorption Using Magnesium Gallate Metal-Organic Framework (Mg-gallate)

**DOI:** 10.3390/molecules28073016

**Published:** 2023-03-28

**Authors:** Marhaina Ismail, Mohamad Azmi Bustam, Nor Ernie Fatriyah Kari, Yin Fong Yeong

**Affiliations:** 1 Carbon Dioxide Research Centre (CO2RES), Universiti Teknologi PETRONAS, Bandar Seri Iskandar 32610, Perak, Malaysia; 2 Centre of Research in Ionic Liquids (CORIL), Universiti Teknologi PETRONAS, Bandar Seri Iskandar 32610, Perak, Malaysia

**Keywords:** adsorption, MOF, Mg-gallate, IAST

## Abstract

Ideal Adsorbed Solution Theory (IAST) is a predictive model that does not require any mixture data. In gas purification and separation processes, IAST is used to predict multicomponent adsorption equilibrium and selectivity based solely on experimental single-component adsorption isotherms. In this work, the mixed gas adsorption isotherms were predicted using IAST calculations with the Python package (pyIAST). The experimental CO_2_ and CH_4_ single-component adsorption isotherms of Mg-gallate were first fitted to isotherm models in which the experimental data best fit the Langmuir model. The presence of CH_4_ in the gas mixture contributed to a lower predicted amount of adsorbed CO_2_ due to the competitive adsorption among the different components. Nevertheless, CO_2_ adsorption was more favorable and resulted in a higher predicted adsorbed amount than CH_4_. Mg-gallate showed a stronger affinity for CO_2_ molecules and hence contributed to a higher CO_2_ adsorption capacity even with the coexistence of a CO_2_/CH_4_ mixture. Very high IAST selectivity values for CO_2_/CH_4_ were obtained which increased as the gas phase mole fraction of CO_2_ approached unity. Therefore, IAST calculations suggest that Mg-gallate can act as a potential adsorbent for the separation of CO_2_/CH_4_ mixed gas.

## 1. Introduction

Single-component and mixed gas adsorption on porous solid materials plays an important role, particularly in the chemical, petrochemical and biochemical industries. The single-component adsorption isotherms (pure gas adsorption isotherms) are typically measured by using commercial instruments with high performance and accuracy. On the other hand, the measurement of multicomponent adsorption equilibrium (mixed gas adsorption isotherms) is one of the most complicated experimental techniques in the adsorption area since it is challenging, time-consuming and requires self-built instruments. Generally, the design and development of adsorptive separation require information of both single-component and multicomponent adsorption equilibria. Therefore, the ability to accurately predict the multicomponent adsorption equilibrium offers a great advantage.

Due to the aforementioned limitations in measuring multicomponent adsorption equilibrium, single-component adsorption isotherms are measured experimentally and used to calculate the mixture behaviors using Ideal Adsorbed Solution Theory (IAST). IAST, developed by Myers and Prausnitz, is a well-studied method to predict multicomponent adsorption equilibrium based only on the experimental data of single-component adsorption isotherms at the same operating temperature [1,2]. In this approach, the adsorbed phase is considered ideal without any interaction in the multicomponent system. Generally, IAST provides reliable predictions of the adsorption and selectivity of a gas mixture and has been used for various solid adsorbents such as zeolites, activated carbon and metal-organic frameworks (MOFs) [3,4,5,6]. Recently, MOFs are considered as emerging porous materials for gas adsorption and separation due to their large surface area and porosity, tunable structure and functionalities [7]. MOF is a classification of inorganic-organic hybrid constituents consisting of metal ions (or clusters) and organic linkers/ligands [8].

Carbon dioxide (CO_2_) and methane (CH_4_) mixture separation is a key challenge for the energy sector and crucial to provide high purity natural gas that meets gas sales specifications. In our previous reported work, the feasibility of gallate-based MOFs for CO_2_ and CH_4_ single-component adsorption was predicted using Grand Canonical Monte Carlo (GCMC) simulation [9]. Among the studied gallate-based MOFs, Mg-gallate showed the highest predicted CO_2_ adsorption capacity and CO_2_/CH_4_ selectivity [9]. The magnesium atom exhibits a significant contribution in the conduction band of states, leading to possible Lewis acidic activity in crystalline materials [10]. This Lewis acidic activity allows a strong interaction with Lewis bases (oxygen in CO_2_). The higher ionic character of the Mg-O bond compared to other metals facilitates stronger charge-quadrupole interaction and degree of polarization between Mg and CO_2_, which favors CO_2_ capture [11,12].

However, there are limited reported studies on the mixed gas adsorption isotherms predicted with IAST calculations at different compositions and temperatures for the CO_2_/CH_4_ mixture using Mg-gallate. Therefore, this work aims to synthesize a magnesium gallate-based MOF (Mg-gallate). Then, the as-synthesized Mg-gallate was further subjected to single-component gas adsorption (static adsorption) of pure CO_2_ and CH_4_. The main objective of this work is to predict the mixed gas adsorption isotherms based on the experimental CO_2_ and CH_4_ single-component adsorption isotherms of Mg-gallate using IAST calculations with the Phyton package at the same operating temperatures. The predicted mixed gas adsorption isotherms were plotted with different compositions of CO_2_/CH_4_ gas mixture at 273, 298 and 313 K, and then used to calculate the corresponding selectivity, which is crucial to evaluate their gas separation performance. These predicted mixed gas adsorption behaviors are expected to give background information for future experimental multicomponent adsorption equilibrium. Understanding the multicomponent adsorption equilibrium is essential for designing adsorption-based separation processes.

## 2. Ideal Adsorbed Solution Theory (IAST)

IAST is a thermodynamic approach in which an ideal solution is considered to be formed by the adsorbed phase, corresponding to Raoult’s law for vapor-liquid equilibrium [3]. To meet the ideal condition, there must be no interaction between the adsorbate molecules in the adsorbed phase, and the spreading pressures of the components must be equal at constant temperature [13]. The highlighted equations for deriving mixed gas isotherms are provided here for convenience, since the detailed explanations of IAST can be found in various sources [3,13,14,15,16]. The spreading pressure can be calculated using the equation below:

(1) $$\frac{\pi A}{RT}=\int_{0}^{P_{i}^{0}}\frac{n_{i}}{P_{i}}{dP}_{i}$$

where π is the spreading pressure, A is the specific surface area of adsorbent (m^2^/g), R is the gas constant (8.314 J K^−1^ mol^−1^), T is the temperature (K) and $n_{i}$ is the adsorption of component i (mmol/g). The partial pressure ($P_{i}$) is defined using an analogue to Raoult’s law:

(2) $$P_{i}=y_{i}P=x_{i}P_{i}^{0}(\pi )(\mathrm{at constant T and \pi})$$

where $P_{i}^{0}(\pi )$ is the partial pressure of pure component *i* calculated at the spreading pressure and temperature of the mixture. $P_{i}$ is the partial pressure of component *i* (bar), P is the total pressure (bar), $y_{i}$ and $x_{i}$ represent mole fractions of component *i* in the gas and adsorbed phases (dimensionless).

The total adsorbed amount ($n_{T}$) can be determined as below:

(3) $$\frac{1}{n_{T}}=\sum_{i=1}^{N}\frac{x_{i}}{n_{i}^{0}}$$

where N is the number of components in the mixture and $n_{i}^{0}$ (mmol/g) is the standard state loading. Therefore, $n_{i}$, which is the adsorption of component i (mmol/g), is calculated using this equation:

(4) $$n_{i}=x_{i}n_{T}$$

The established adsorption selectivity based on IAST is defined by the following equation:

(5) $$S_{ij}=\frac{x_{i}/x_{j}}{y_{i}/y_{j}}$$

## 3. Result and Discussions

**Figure 1:**
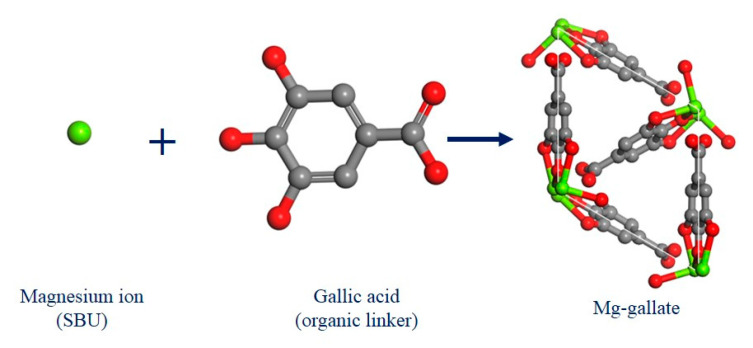
The structures of building units and Mg-gallate.

Mg-gallate with the chemical formula of Mg(C_7_O_5_H_4_)·2H_2_O is a type of crystal structure that contains magnesium ions as the secondary building unit (SBU) connected with oxygen atoms of gallic acid (organic linker) to form a three-dimensional framework [17]. The pore structure of Mg-gallate creates spaces or channels depending on its pore size within the framework that can accommodate the other molecules, in this case CO_2_ or CH_4_ molecules. The pore size of Mg-gallate can vary depending on the synthesis method and conditions used. Generally, the pore size ranges from a few amstrongs to nanometers. Its unique structure and pore size made it a promising CO_2_ and CH_4_ adsorbent. Figure 1 shows the structures of the building units and the resulting framework drawn using Material studio. Green, gray and red represent magnesium, carbon and oxygen respectively, while hydrogen atoms are omitted for clarity.

### 3.1. Characterization Analysis

#### 3.1.1. Powder X-ray Diffraction (PXRD) Pattern

**Figure 2:**
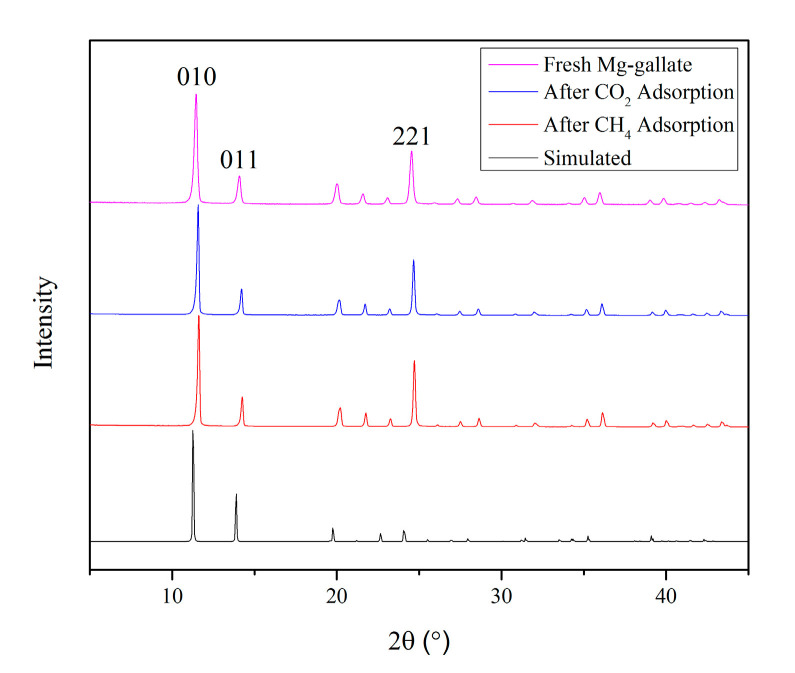
PXRD patterns of Mg-gallate.

The PXRD patterns of Mg-gallate were performed and are shown in Figure 2 to confirm its crystalline nature.

The fresh Mg-gallate (as-synthesized) exhibited a PXRD pattern with three significant diffraction peaks around 11.35°, 14.05° and 24.53° that corresponded to Miller indices (*hkl*) values of 010, 011 and 221, respectively, which showed that this porous material was in a good agreement with the previous reported work [18]. The signature of crystallinity of Mg-gallate could be detected by the sharp peaks. The average crystallite size for Mg-gallate was calculated to be 34.9 nm, which could be determined through X-ray diffraction line broadening by using the Debye-Scherrer formula [19]. There is a limited source of gallate-based MOFs in the Joint Committee on Powder Diffraction Standards (JCPDS) database and the highest peak search score obtained by using X’Pert HighScore Plus software was 55 with a reference number of 96-433-5642.

On the other hand, the diffraction peaks of fresh Mg-gallate almost overlapped with those of spent Mg-gallate. Generally, XRD pattern originates from the bulk rather than the surface of a material. CO_2_ and CH_4_ adsorption was classified as physisorption (physical adsorption), in which the molecules are attached to the surface of Mg-gallate due to a weak force known as Van der Waals force [20]. In addition, physisorption was not followed by incorporation into the crystal structure, which was confirmed by the unaffected XRD patterns between fresh Mg-gallate and after CO_2_ and CH_4_ adsorption.

The XRD pattern of the as-synthesized Mg-gallate exhibited peaks at around 11.35°, 14.05°, 20.03°, 21.58°, 23.08°, 24.53°, 25.97°, 27.31°, 28.50°, 31.92°, 35.05°, 36.01°, 39.06°, 39.88°, 42.41° and 43.30°, which well-agreed with the simulated pattern. The simulated pattern was calculated based on the reference structure in the Cambridge Crystallographic Data Centre (CCDC) with the database identifier of GELVEZ and deposition number of 286498. However, the intensity of the peaks between the as-synthesized and the simulated was different due to the elements of the as-synthesized Mg-gallate might not be uniformly distributed throughout the crystal structure. Typically, the intensity of the diffraction peaks is directly proportional to the amount of elements present in the material [21]. In addition, the as-synthesized peaks were broader compared to the simulated peaks since the simulated crystallite size was calculated to be 46.4 nm. Broader peaks indicate a smaller size of crystallite [21].

#### 3.1.2. Fourier Transform Infrared (FTIR) Spectrum

**Figure 3:**
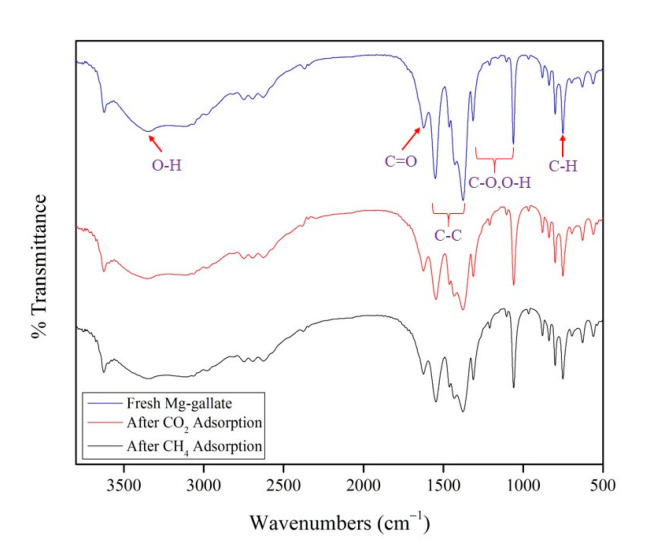
FTIR spectra of Mg-gallate.

The FTIR spectra of Mg-gallate with the patterns that provide structural insights are illustrated in Figure 3.

For fresh Mg-gallate, a strong and broad band in the IR spectrum in the region of 3500–2800 cm^−1^ was found to be the stretching vibration of the O-H of a carboxyl group. The strong and narrow peak at 1622 cm^−1^ represented by a carbonyl (C=O) indicated the presence of a carboxyl group in the gallic acid. Three peaks located at 1554, 1462 and 1376 cm^−1^ were typical stretching vibrations of C-C bonds in an aromatic ring of the gallic acid. There were a few peaks in the region 1300–1000 cm^−1^ which indicated the stretching vibrations of C-O bonds and the bending vibration of O-H bonds in the aromatic ring of the gallic acid. C-H bonds of the aromatic ring were located at 748 cm^−1^. Therefore, the IR spectrum identified the functional groups present in the structure of Mg-gallate. There is a very good agreement between the band positions of this work and the literature [19]. On the other hand, the IR spectra also displayed peaks identical to those of the spent Mg-gallate, which confirmed that CO_2_ and CH_4_ adsorption did not compromise the structure of Mg-gallate.

#### 3.1.3. Thermogravimetric Analysis (TGA)

**Figure 4:**
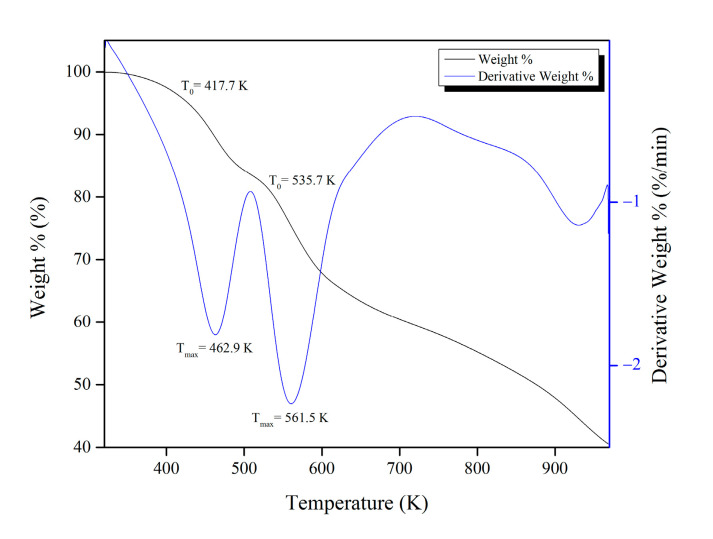
TGA curve of Mg-gallate.

Figure 4 shows the TGA curve to describe the thermal stability of Mg-gallate.

The thermal stability of Mg-gallate is reported in the form of onset temperature (T_0_) and decomposition temperature (T_max_). T_0_ is obtained from the intersection of the baseline and the tangent of the sample weight against the temperature, while T_max_ is the temperature at the point where the maximum weight loss of the sample occurs [22]. Based on Figure 4, the Mg-gallate started to decompose at T_0_ = 417.7 K, which indicated the beginning of the first stage of weight loss. T_max_ for the first stage occurred at 462.9 K when 10.5% was lost due to the release of the remaining water and ethanol. Meanwhile, the decomposition of the gallic acid (organic linker) started at the second stage of the weight loss, at T_0_ = 535.7 K. Approximately 24.9% of Mg-gallate was reported as the maximum weight loss at T_max_ = 561.5 K. It also showed that the structure of Mg-gallate was stable up to 561.5 K. Therefore, Mg-gallate can stand and operate at high-temperatures.

#### 3.1.4. Porous Properties

**Figure 5:**
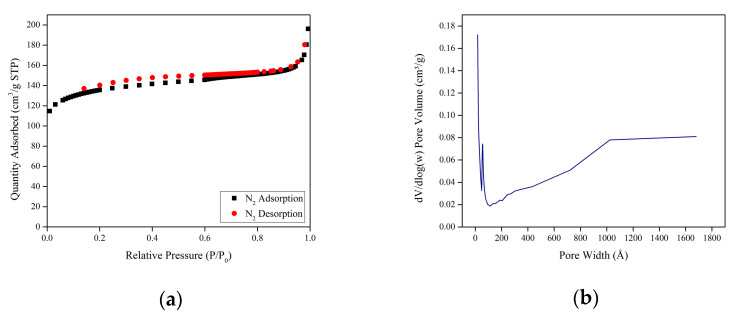
(**a**) N_2_ adsorption-desorption isotherms; (**b**) BJH pore size distribution of Mg-gallate.

Figure 5 shows the adsorption-desorption isotherms of nitrogen (N_2_) and pore size distribution. At the initial stage, a rapid increment in N_2_ uptake was observed, followed by a plateau phase, which represented the monolayer adsorption. Due to the further adsorption, a multilayer was produced. In addition, the existence of a small hysteresis loop in the N_2_ adsorption-desorption curves could be seen at the region P/P_0_ 0.15–0.7. This is due to capillary condensation in which gas adsorbed in pores at low density, spontaneously condenses into a liquid-like state inside the pores of a framework. In other words, hysteresis occurs when desorption does not occur in the same way as adsorption.

The Brunauer–Emmett–Teller (BET) equation was used to calculate the specific surface area in the relative pressure range of 0.00–0.09. The BET surface area of Mg-gallate was found to be 512.38 m^2^/g. Furthermore, the pore volume was estimated using the t-plot method and the value was 0.187 cm^3^/g.

The classification of porous materials is usually based on the diameter of their pores, microporous (<2 nm), mesoporous (2–50 nm) and macroporous (>50 nm) according to the International Union of Pure and Applied Chemistry (IUPAC) [23]. Due to the nature and mode of MOFs’ preparation, MOFs generally have a non-uniform range of pores, hence an average pore size is used. The average pore size of Mg-gallate is 6.66 nm and it is classified as mesoporous (2–50 nm). The pore size distribution was interpreted based on the relationship between the pore width (w) and dV/dlog(w) using the Barrett-Joyner–Halenda (BJH) model [24]. The porous properties of Mg-gallate are tabulated in Table 1. Given its porous properties, Mg-gallate was expected to exhibit a remarkable performance in CO_2_/CH_4_ adsorptive separation.

### 3.2. Single-Component Gas Adsorption

An equilibrium distribution is reached if the adsorbent and guest molecules come into contact and can be explained quantitatively. The equilibrium behavior is expressed in terms of the amount of adsorbate as a function of partial pressure at a fixed temperature. This equilibrium can be illustrated in what is called an isotherm. Adsorption isotherms are important for the description of how the adsorbate will interact with the adsorbent based on the pore surface properties and affinity, which results in the adsorption capacity. Single-component gas adsorption capacity is determined from the pure gas sorption isotherm at a certain temperature and pressure. The adsorption capacity is an important parameter in the evaluation of MOFs for CO_2_ capture and is the main evaluation tool for gas capture applications.

**Figure 6:**
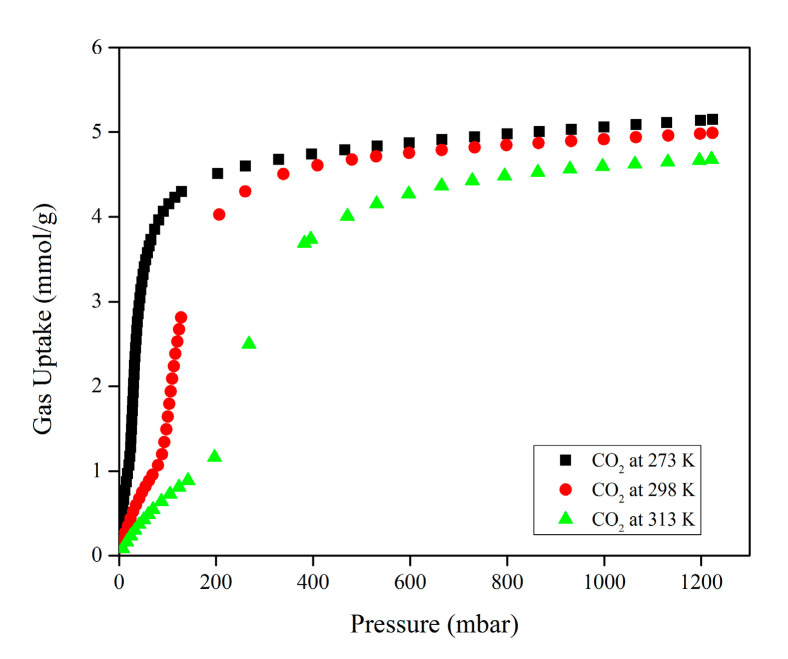
Experimental CO_2_ single-component adsorption isotherms of Mg-gallate at 273, 298 and 313 K.

In order to investigate the potential impact of the porous nature of Mg-gallate on CO_2_ and CH_4_ behaviors, single-component gas adsorption was conducted at three different temperatures. Figure 6 illustrates the experimental CO_2_ single-component adsorption isotherms at three different temperatures with S-shaped isotherms (sigmoidal).

The amount of CO_2_ adsorbed on Mg-gallate increased as the pressure increased for all temperatures. However, the isotherms decreased as the temperatures increased from 273 to 313 K. The reason behind is that the surface adsorption energy and the molecular diffusion rate increase with the temperature, causing the gas molecules to unsteadily move and making it difficult for them to attach to the surface of the adsorbent [25]. Subsequently, a higher temperature condition is not advantageous for the adsorption process and may cause a reduction in the adsorbed amount. This phenomenon in which the adsorbed amount increases with pressure and decreases with temperature is due to Le Chatelier’s principle [26]. According to Le Chatelier’s principle, the exothermic process at higher temperature prefers conditions that produce less heat. In addition, since adsorption is known as an exothermic process, it is expected to have a higher adsorbed amount at a lower temperature due to a higher affinity between the adsorbent and the adsorbate, which results in the adsorbent’s surface being covered with more adsorbates.

The influence of the porous nature of Mg-gallate on the CO_2_ adsorption capacity could be observed at 1 bar, showing that Mg-gallate offered a CO_2_ adsorption capacity of 5.06, 4.92 and 4.60 mmol/g at 273, 298 and 313 K, respectively. MOFs are considered competitive adsorbents once they can offer a CO_2_ adsorption capacity of 3.0 mmol/g or higher [27].

**Figure 7:**
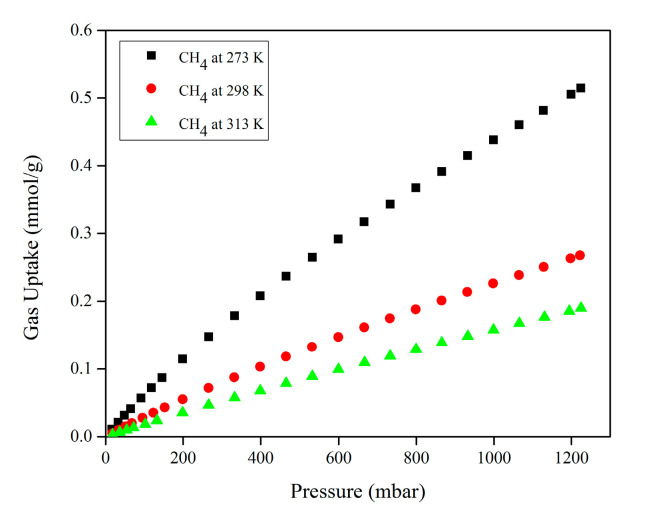
Experimental CH_4_ single-component adsorption isotherms of Mg-gallate at 273, 298 and 313 K.

Figure 7 illustrates the experimental CH_4_ single-component adsorption isotherms at three different temperatures.

The experimental CH_4_ single-component adsorption isotherms also displayed the same pattern in which the amount of CH_4_ adsorbed on Mg-gallate increased with increasing pressure for all temperatures with a linear shape isotherm. However, the isotherms decreased as the temperature increased from 273 to 313 K. The linear shape isotherm is also known as Henry adsorption isotherm. Henry isotherm is considered the simplest adsorption isotherm since the partial pressure of adsorptive gas corresponds to the amount of adsorbate [28]. Based on the isotherms, the equilibrium adsorbed amount of CH_4_ on Mg-gallate was substantially proportional to the gas partial pressure. Mg-gallate provided a CH_4_ adsorption capacity of 0.438, 0.226 and 0.158 mmol/g at 273, 298 and 313 K, respectively and at 1 bar. It is worth noting that Mg-gallate is able to prevent CH_4_ from being adsorbed because of low gas uptake due to poor affinity between the framework and CH_4_ molecules.

The CO_2_ adsorption capacity of Mg-gallate is greater than that of CH_4_ under the same condition due to the thermodynamic equilibrium effect. The thermodynamic equilibrium effect arises due to the difference in the affinity/interaction of various gas molecules to be adsorbed on the adsorbent surface. The affinity between the adsorbent and the adsorbate depends on the different physical properties of the gas molecules, such as polarizability and quadrupole moment. This large gap between the adsorption capacity values of CO_2_ and CH_4_ verified that CO_2_ is a strong adsorbate and adsorbs more favorably on Mg-gallate due to a stronger interaction between CO_2_ and Mg-gallate. It is known that CO_2_ has a larger polarizability (29.1 × 10^−25^ cm^3^ for CO_2_, 25.9 × 10^−25^ cm^3^ for CH_4_) and quadrupole moment (4.30 × 10^−26^ esu cm^2^ for CO_2_, 0 for CH_4_) compared to CH_4_ [29]. The higher the polarizability of the adsorbate, the higher the interaction with the adsorbent surface. This interaction is favorable since the quadrupole moment of CO_2_ is complementary to the polarization of Mg-gallate. The CO_2_ adsorption capacity was greatly affected by the interactions of Mg-gallate and CO_2_ molecules, which occurred at two binding sites. The CO_2_ molecules firstly occupied the open metal sites of the secondary building unit (SBU) and then interacted with the organic linker. Based on this phenomenon, the separation of the CO_2_/CH_4_ mixture is feasible.

### 3.3. Isotherm Models

The experimental single-component adsorption isotherms of CO_2_ and CH_4_ should be first fitted using a proper model in order to perform the integration required by IAST. It is to discrete data so that the uncertainty in the multicomponent predictions always comes from this fit of data [3]. There are no restrictions on the choice of the isotherm models as long as they can fit precisely. Phyton can offer several isotherm models such as the Langmuir, quadratic, BET, Henry, approximated Temkin, and dual-site Langmuir to fit the experimental single-component adsorption isotherms [15]. The model parameters are tabulated in Table 2.

**Figure 8:**
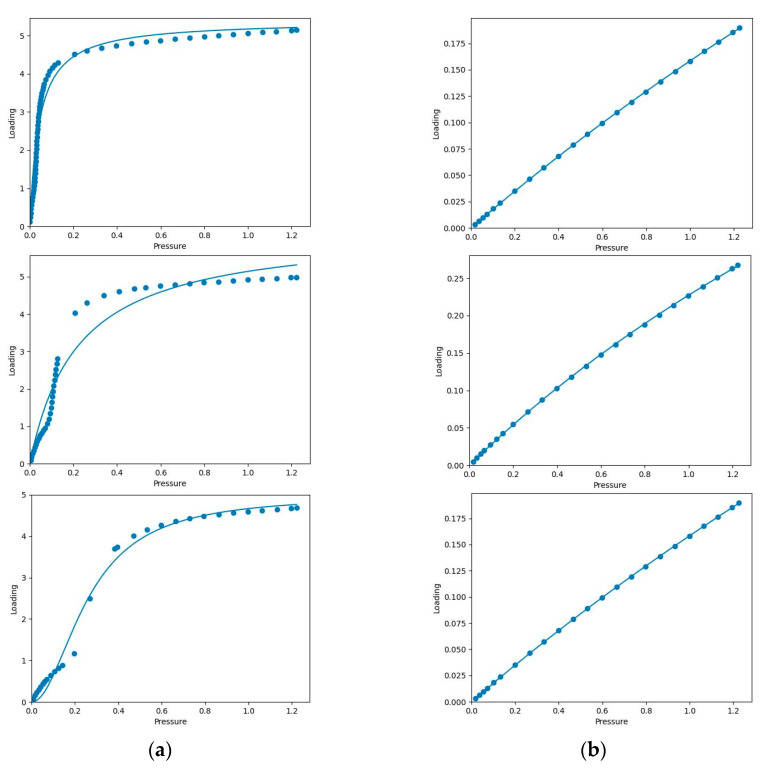
Experimental single-component adsorption isotherms (dot) and Langmuir fitted-isotherms (line): (**a**) CO_2_; (**b**) CH_4_ at 273, 298 and 313 K, respectively.

The differences between the studied isotherm models appeared as a function of how accurately the models fit the experimental single-component adsorption isotherms. It is important to emphasize that if IAST is used using a poorly fitted model, then the multicomponent prediction is expected to be inaccurate even though IAST itself is accurate for the system of interest. According to Table 2, the Langmuir model gave the best fit for all single-component adsorption isotherms of CO_2_ and CH_4_ at 273-313 K in terms of RMSE value with relevant parameter values. Figure 8 shows the experimental single-component adsorption isotherms and Langmuir fitted-isotherms of Mg-gallate for CO_2_ and CH_4_ at three different temperatures in terms of loading (mmol/g) versus pressure (bar).

The Langmuir model is a model to describe the adsorption behavior of gases and is defined as below [30,31]:

(6) $$q_{e}=\frac{MK_{L}P_{e}}{1+K_{L}P_{e}}$$

where, $q_{e}$ (mmol/g) represents the amount of the adsorbed gas per unit mass of adsorbent at equilibrium, M (mmol/g) is the maximum adsorption capacity, $K_{L}$ (1/bar) is the Langmuir constant related to the free energy of adsorption and $P_{e}$ (bar) is the equilibrium pressure.

It is a widely used isotherm model due to its simplicity, effectiveness and reasonable explanation of its parameters [32]. The Langmuir model was developed based on the assumption of monolayer adsorption, no interactions between adsorbed molecules, equal energy of adsorption and molecules adsorbed at fixed sites that do not migrate over the surface [33].

Based on Table 2, the maximum adsorption capacity (M) values of CO_2_ molecules on Mg-gallate increased as the Langmuir constants ($K_{L}$) decreased from 273 to 313 K. $K_{L}$ explains the adsorption energy and the affinity between the adsorbates and the adsorption sites of the adsorbent in whose reduction of this factor causes the increase of M [34]. Normally, $K_{L}$ decreases with increasing temperature since adsorption is an exothermic process. All the values of $K_{L}$ for CO_2_ are much higher than those for CH_4_, resulting in higher M values for CO_2_. Meanwhile, the values of M and $K_{L}$ for CH_4_ fluctuated.

### 3.4. Prediction of CO_2_ and CH_4_ by IAST Calculations

**Figure 9:**
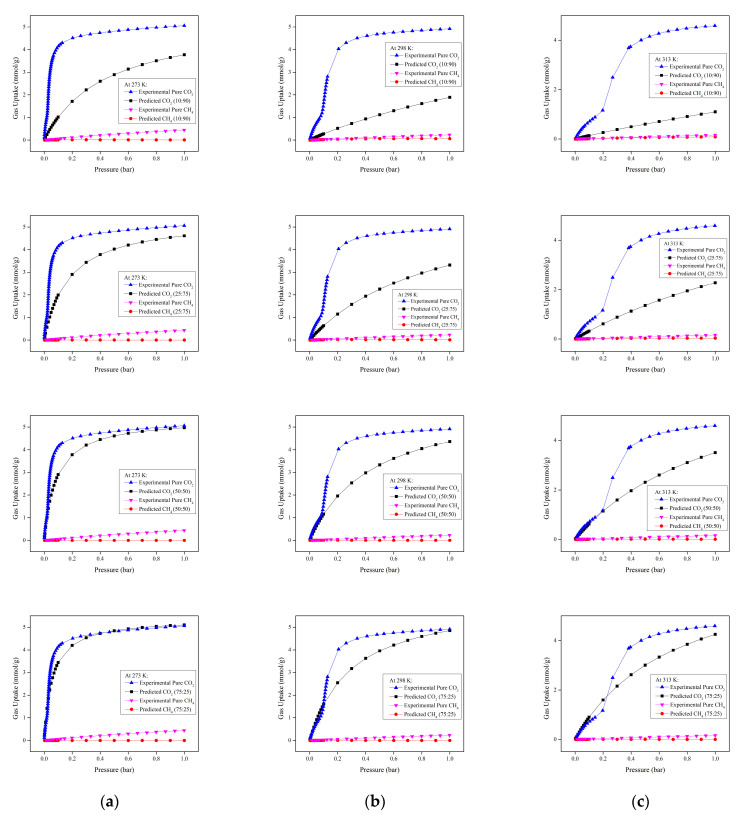
Experimental (single-component) and predicted (multicomponent) adsorption isotherms of CO_2_ and CH_4_ for 10:90. 25:75, 50:50 and 75:25 compositions at: (**a**) 273 K; (**b**) 298 K and (**c**) 313 K, respectively.

Based on the experimental single-component adsorption isotherms, the multicomponent adsorption isotherms predicted using IAST with the Phyton package for the CO_2_/CH_4_ gas mixture for different compositions at 273, 298 and 313 K and up to 1 bar are shown in Figure 9.

Figure 9 shows that the predicted amount of adsorbed CO_2_ and CH_4_ on Mg-gallate is lower than the experimental amount. This is due to competitive adsorption between the different components of the gas mixture in which the CO_2_ and CH_4_ molecules competed with each other for the adsorption sites. Consequently, the presence of CH_4_ in the gas mixture slightly affected the adsorption of CO_2_ on Mg-gallate. Nevertheless, CO_2_ was still dominant and favorably adsorbed compared to CH_4_ due to a stronger interaction between CO_2_ and Mg-gallate, resulting in a lower predicted amount of CH_4_ due to the previously mentioned thermodynamic equilibrium effect. The predicted CO_2_ adsorption isotherms exhibited the same pattern as the experimental single-component adsorption isotherms in that they increased with pressure but decreased as temperatures increased from 273 to 313 K. In addition, it is worth noting that the predicted adsorbed amount of CO_2_ increased as the composition of CO_2_ in the CO_2_/CH_4_ gas mixture increased. Moreover, the predicted amount of CH_4_ is too low and almost showed a plateau pattern. Indeed, the multicomponent adsorption isotherms using IAST led to the evaluation of the capability of Mg-gallate.

At initial stage of adsorption (lower pressure range), the CO_2_ isotherms demonstrated a rapid increase in gas uptake, especially at a higher gas-phase mole fraction of CO_2_ in which the isotherms became steeper but less steep as temperature increased. It means that the fastest initial stage of adsorption was achieved by the 75:25 composition adsorption at 273 K since it formed the steepest curve in the lowest pressure range. In addition, the steep curve was followed by the plateau gradient, indicating the saturation of the monolayer, which proved the occurrence of the gas adsorption limit. It is worth noting that the predicted CO_2_ adsorption isotherms fulfilled the characteristics and belonged to Type I, while the CH_4_ adsorption isotherms almost corresponded to the linear isotherms.

**Figure 10:**
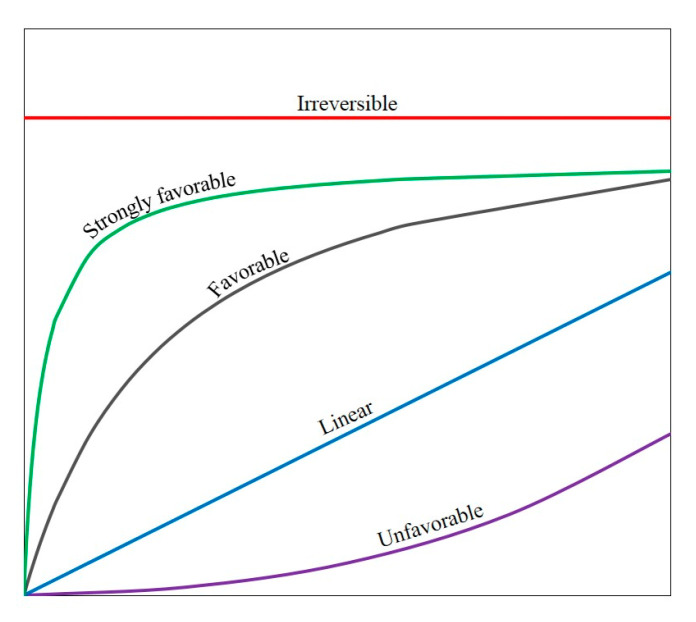
Nature of the adsorption isotherms [35].

The isotherm shape can be used to determine whether an adsorption system is favorable or unfavorable. Based on the isotherm shapes, the predicted CO_2_ adsorption systems were more favorable as the gas-phase mole fraction of CO_2_ approached unity and at lower temperature. In addition, the predicted CH_4_ adsorption systems could be considered approached unfavorable. The isotherm shapes in this work followed the pattern mentioned by the literature as shown in Figure 10 [35].

The degree of favorability in this work was supported by the important feature of the Langmuir isotherm that can be expressed in terms of a dimensionless constant separation factor or equilibrium parameter ($R_{L}$). $R_{L}$ represents the isotherm shape and the nature of the adsorption. It can be defined as below [36]:

(7) $$R_{L}=\frac{1}{1+K_{L}P_{e}}$$

**Figure 11:**
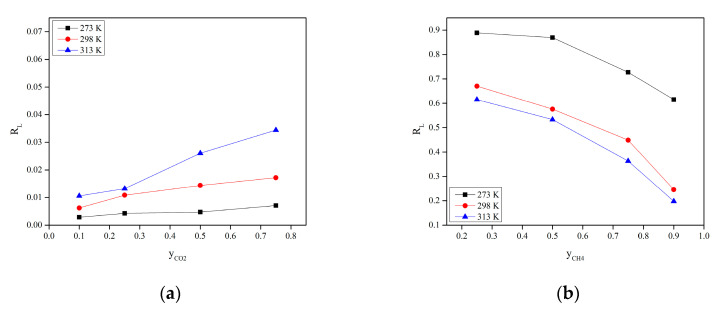
R_L_ of: (**a**) CO_2_; (**b**) CH_4_ at 273, 298 and 313 K.

The value of $R_{L}$ determines whether the nature of the isotherm is irreversible ($R_{L}$ = 0), favorable (0 < $R_{L}$ < 1), linear ($R_{L}$ = 1) or unfavorable ($R_{L}$ > 1). The values of $R_{L}$ for different CO_2_/CH_4_ compositions at different temperatures are illustrated in Figure 11. The values of $R_{L}$ in the range of 0–1 verified that CO_2_ adsorption was favorable. In addition, CO_2_ adsorption was less reversible in all CO_2_ compositions, confirmed by the lower values of $R_{L}$. Furthermore, CH_4_ adsorption was less favorable based on higher values of $R_{L}$ compared to CO_2_. Additionally, the highest values of $R_{L}$ for CH_4_ that approached unity (reversible) represented the ease of adsorbed CH_4_ molecules being desorbed from Mg-gallate. This is the reason behind the lower CH_4_ uptake compared to CO_2_. In short, the degree of favorability is moving towards zero, indicating the completely ideal irreversible process than unity, meaning a completely reversible process [36].

### 3.5. IAST Selectivity of CO_2_ and CH_4_

**Figure 12:**
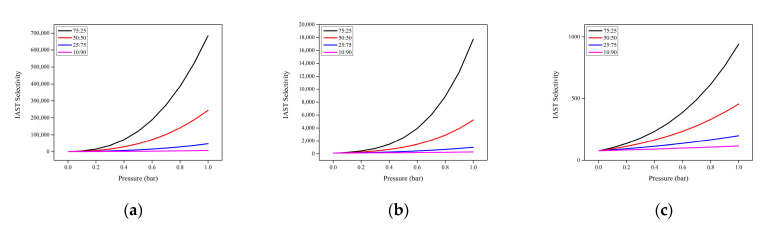
IAST selectivity of CO_2_ and CH_4_ at: (**a**) 273 K; (**b**) 298 K and (**c**) 313 K, respectively.

The IAST method is also applicable to calculate the mixed gas selectivity (IAST selectivity) [37]. Adsorption selectivity occurs due to the difference in affinity of the various components of the gas mixture to be adsorbed on the pore surface of adsorbents. Selectivity can be simply understood as the affinity of Mg-gallate to adsorb CO_2_ compared to CH_4_. The adsorption selectivity established based on IAST calculations are shown in Figure 12.

The high IAST selectivity values for CO_2_/CH_4_ represented that CO_2_ was a strong adsorbate and adsorbed more favorably on Mg-gallate compared to CH_4_. A high selectivity for CO_2_ over the other components of a gas mixture is necessary in CO_2_ capture applications. The IAST selectivity increased rapidly as the gas-phase mole fraction of CO_2_ approached unity. However, it could be said that IAST overpredicted the selectivity of the CO_2_/CH_4_ mixture, especially at 273 and 298 K. It usually happens because one component (CO_2_) strongly adsorbed compared to the other component (CH_4_). Furthermore, uncertainty arose as IAST was applied in regimes that required extrapolation beyond the experimental data. Despite these drawbacks, IAST is known as the established standard in multicomponent adsorption predictions [3].

As the temperature decreased, the IAST selectivity increased, confirming that Mg-gallate performed better at low temperature for both single-component and mixed gas adsorption. Adsorption is an exothermic process in which low temperature conditions are favored. Since adsorption is inversely proportional to temperature, the adsorbate molecules tend to desorb from the adsorbent under high temperature conditions, and this process is called desorption. This is the reason behind lower adsorption uptake under high temperature conditions. The IAST selectivity of Mg-gallate in this work is compared to the other gallate-based MOFs at 298 K and 1 bar for CO_2_/CH_4_ composition of 50:50. This Mg-gallate could offer an IAST selectivity of 5236, higher than Ni-gallate (3171), Mg-gallate (2497) and Co-gallate (198) under the same operating conditions, done in previous work [38].

Even at high content of CH_4_ (low content of CO_2_), Mg-gallate could still offer the potential IAST selectivity. The composition of CH_4_ in natural gas is normally in the range of 75–98 vol% [39]. Therefore, these outcomes are relevant to the practical challenges of natural gas purification and Mg-gallate can be considered as the promising adsorbent for CO_2_/CH_4_ separation.

## 4. Materials and Methods

### 4.1. Materials

Magnesium (II) chloride anhydrous, MgCl_2_ (98%), gallic acid anhydrous, C_7_H_6_O_5_ (98%), potassium hydroxide, KOH (85%), and ethanol absolute, C_2_H_5_OH (99.8%) were obtained from Merck without any further purification. CO_2_ (99.99%) and CH_4_ (99.995%) purified grade gas tanks were obtained from Linde Malaysia.

### 4.2. Synthesis of Mg-Gallate

Mg-gallate was prepared by hydrothermal synthesis according with previously reported work [17]. 50 mmol of MgCl_2_ and 100 mmol of gallic acid were added to 250 mL of 0.5 M KOH aqueous solution in a round-bottomed flask. Then, the mixture was heated and refluxed at 80 °C and ambient pressure with continuous stirring for 24 h. After being naturally cooled, the product was collected and washed two times with deionized water. Then, the product was immersed in ethanol with double replenishment.

### 4.3. Characterization of Mg-Gallate

The Powder X-ray Diffraction (PXRD) pattern for Mg-gallate was recorded using X’Pert Powder Panalytical equipped with Cu Kα radiation (λ = 1.54 Å). The data was collected in the range of 5–50° (2θ angle range) with a scan step size of 0.02°.

The Fourier Transform Infrared Spectroscopy (FTIR) spectrum was performed with a potassium bromide flakelet (KBr) method using a Thermo Scientific Nicolet IS5 in the wavenumber range of 500–4000 cm^−1^ with 16 scans. Background scanning was performed to determine the presence of air impurities such as the carbon dioxide peak.

The Thermogravimetric Analysis (TGA) of Mg-gallate was measured using a Perkin Elmer STA 6000. Approximately 5.0 mg of sample was weighed in a crucible pan and placed on the sample holder. The measurement was conducted at a 283 K/min heating rate under a temperature range of 323–973 K under N_2_ condition.

The porous properties of Mg-gallate were investigated via N_2_ adsorption-desorption isotherms measured at 77 K using 3FLEX Micromeritics Surface Characterization.

For every characterization analysis, Mg-gallate was degassed at 393 K for 24 h under an ultrahigh vacuum.

### 4.4. Single-Component Gas Adsorption

Prior to the adsorption measurement, Mg-gallate was first degassed at 393 K for 24 h under an ultrahigh vacuum. The single-component isotherms for both CO_2_ and CH_4_ were measured at 273, 298 and 313 K using 3FLEX Micromeritics Surface Characterization.

### 4.5. IAST Calculation

The mixed gas adsorption isotherms were predicted from experimental CO_2_ and CH_4_ single-component adsorption isotherms using IAST calculations with the Python package (pyIAST), as studied in previous work [15]. The detailed methodology was explained there, here the highlighted points were simplified.

First, pyIAST characterized the experimental CO_2_ and CH_4_ single-component adsorption isotherms by fitting the suitable analytical models such as the Langmuir, quadratic, BET, Henry, approximated Temkin, and Dual-site Langmuir to the data. Second, pyIAST linearly interpolated the data. Finally, pyIAST performed IAST calculations to predict the mixed gas adsorption isotherms. The compositions of the CO_2_/CH_4_ mixed gas were varied to 10:90, 25:75, 50:50 and 75:25. From the mixed gas adsorption isotherms, the IAST selectivity was calculated.

## 5. Conclusions

IAST is a method to describe the multicomponent adsorption equilibrium in which adsorption selectivity can be predicted solely based on experimental single-component adsorption isotherms. The IAST calculation came with model fittings of the experimental data where the Langmuir model gave the best fit based on the RMSE value with relevant parameter values. The predicted amount of adsorbed CO_2_ and CH_4_ on Mg-gallate is lower than experimental amount due to competitive adsorption among the different components of the gas mixture. The stronger interaction between CO_2_ and Mg-gallate contributed to the higher predicted adsorbed amount of CO_2_ than CH_4_, which increased as the compositions of CO_2_/CH_4_ increased. It is also confirmed that the CO_2_ adsorption was favorable based on the values of $R_{L}$. IAST selectivity increased rapidly as the gas-phase mole fraction of CO_2_ approached unity. Therefore, Mg-gallate is a promising adsorbent for separation of CO_2_/CH_4_ based on IAST calculations. In summary, the main purpose of this work was achieved that is using IAST calculations to predict the potential selectivity of Mg-gallate based on simple measurements of single-component adsorption isotherms, since selectivity is the important factor when working with multicomponent gas adsorption. These predicted multicomponent adsorption behaviors can be applied in the design of practical gas adsorption and separation processes.

## Figures and Tables

**Table 1:** Porous properties of Mg-gallate.

BET Surface Area (m^2^/g)	Pore Volume (cm^3^/g)	BJH Pore Size (nm)
512.38	0.187	6.66

**Table 2:** Parameter values of isotherm models.

Model	Parameter		CO_2_			CH_4_	
		273 K	298 K	313 K	273 K	298 K	313 K
Langmuir	M	5.3934	6.2675	7.2937	1.4239	1.1525	1.4239
	K_L_	23.45	4.57	1.87	0.1251	0.2456	0.1251
	RMSE	0.3321	0.3882	0.3825	4.41 × 10^−4^	8.80 × 10^−4^	4.41 × 10^−4^
Quadratic	M	2.4819	2.5036	2.4885	5.25 × 10^−4^	5.25 × 10^−4^	5.25 × 10^−4^
	K_a_	5.52	−0.4963	−0.0225	5.0 × 10^−4^	5.0 × 10^−4^	5.0 × 10^−4^
	K_b_	768.40	60.69	14.79	5.0 × 10^−4^	5.0 × 10^−4^	5.0 × 10^−4^
	RMSE	0.1586	0.1953	0.1961	0.1094	0.1546	0.1094
BET	M	6.1832	5.25 × 10^−4^	5.25 × 10^−4^	5.25 × 10^−4^	5.25 × 10^−4^	5.25 × 10^−4^
	K_a_	18.53	5.0 × 10^−4^	5.0 × 10^−4^	5.0 × 10^−4^	5.0 × 10^−4^	5.0 × 10^−4^
	K_b_	−0.1666	5.0 × 10^−4^	5.0 × 10^−4^	5.0 × 10^−4^	5.0 × 10^−4^	5.0 × 10^−4^
	RMSE	0.3100	3.2372	3.2059	0.1094	0.1546	0.1094
Henry	K_H_	6.1555	5.7421	5.0373	0.1591	0.2296	0.1591
	RMSE	2.1951	1.3555	0.8717	3.10 × 10^−3^	8.03 × 10^−3^	3.10 × 10^−3^
Approximated Temkin	M	4.7234	2.6270	5.25 × 10^−4^	1.3694	1.6112	1.3694
	K_T_	15.59	3.74	5.0 × 10^−4^	0.1301	0.1766	0.1301
	θ	−1.9074	−8.1530	5.0 × 10^−4^	−0.0473	0.4733	−0.0473
	RMSE	0.2335	0.2330	3.2059	4.46 × 10^−4^	8.0 × 10^−4^	4.46 × 10^−4^
Dual-site Langmuir	M_1_	0.5384	5.25 × 10^−4^	4.4854	5.25 × 10^−4^	5.25 × 10^−4^	5.25 × 10^−4^
	K_1_	23.45	5.0 × 10^−4^	1.8707	5.0 × 10^−4^	5.0 × 10^−4^	5.0 × 10^−4^
	M_2_	4.8550	5.0 × 10^−4^	2.8083	5.0 × 10^−4^	5.0 × 10^−4^	5.0 × 10^−4^
	K_2_	23.45	5.0 × 10^−4^	1.8707	5.0 × 10^−4^	5.0 × 10^−4^	5.0 × 10^−4^
	RMSE	0.3321	3.2372	0.3825	0.1094	0.1546	0.1094

## Data Availability

The raw and processed data required to reproduce these findings cannot be shared as the data is part of the ongoing study.

## Sample Availability

Samples of the compounds are available from the authors.
